# Assesment of radiotherapy effects on the blood flow in gingiva and dental pulp - a laser Doppler flowmetry study

**DOI:** 10.1590/1678-7757-2022-0329

**Published:** 2022-12-02

**Authors:** Svetlana Antic, Biljana Markovic-Vasiljkovic, Bojan Dzeletovic, Drago B. Jelovac, Jovana Kuzmanovic-Pficer

**Affiliations:** 1 University of Belgrade School of Dental Medicine Center for Radiological Diagnostics Belgrade Serbia University of Belgrade, School of Dental Medicine, Center for Radiological Diagnostics, Belgrade, Serbia.; 1 University of Belgrade School of Dental Medicine Department of Restorative Odontology and Endodontics Belgrade Serbia University of Belgrade, School of Dental Medicine, Department of Restorative Odontology and Endodontics, DentalNet Research Group, Belgrade, Serbia.; 2 University of Belgrade School of Dental Medicine Clinic for Maxillofacial Surgery Belgrade Serbia University of Belgrade, School of Dental Medicine, Clinic for Maxillofacial Surgery, Belgrade, Serbia.; 3 University of Belgrade School of Dental Medicine Department for Medical Statistics and Informatics Belgrade Serbia University of Belgrade, School of Dental Medicine, Department for Medical Statistics and Informatics, Belgrade, Serbia.

**Keywords:** Blood flow, Dental pulp, Gingiva, Radiotherapy, Laser Doppler flowmetry

## Abstract

**Objective:**

This study aims to determine and compare the dental pulp and gingival blood flow in patients referred for oropharyngeal radiotherapy (RT) at three different time points: before the start, immediately after, and six months following the completion of RT. The aim is also to evaluate the dependence of the pulp and gingival blood flow on the radiation dose.

**Methodology:**

A prospective study included 10 patients referred for intensity-modulated RT (IMRT) in the oropharyngeal region, with at least one intact tooth surrounded by a healthy gingiva. The dose received by each selected tooth and adjacent gingiva was determined according to the map of treatment planning and computer systems. The blood flow measurements were performed using the laser Doppler flowmetry (LDF) method.

**Results:**

Comparing vascular flows at three different time points, the median blood flow in the dental pulp showed no statistically significant difference (p=0.325), contrary to gingiva (p=0.011). Immediately after RT completion, the gingival flow significantly increased compared to its starting point (p=0.012). The pulp flow correlated negatively with the radiation dose, whereas a strong correlation was noted 6 months following the RT completion.

**Conclusions:**

RT caused a significant acute gingival blood flow increase, followed by a long-term (over six months) tendency to return to the starting levels. The dental pulp blood flow is differently affected by higher radiation doses (over 50Gy) in comparison to lower doses (below 50Gy). During RT planning, considering the possibility of protecting the teeth localized near the Gross Tumor Volume as a sensitive organ is recommended.

## Introduction

Currently, radiotherapy (RT) is an integral part of the treatment of various neoplastic processes. Applied in the treatment of oral and oropharyngeal malignancies, RT can be responsible for many transient or permanent side effects in the oral cavity, such as oral mucositis, radiation caries, osteoradionecrosis, or alteration of salivary gland function.^1,2^ By introducing 3D conformal planning and intensity-modulated radiotherapy (IMRT), surrounding healthy organs became more protected from its harmful effects.^2^ Studies confirm greater sparing of the salivary glands and a lower rate of xerostomia when using modern RT modalities.^2-4^ However, teeth with supporting tissues, located within the irradiated volume, are exposed to harmful effects to a greater or lesser extent, depending on the individual dose they received, which can differ from the tumor dose. RT also causes inflammatory changes in the oral mucosa and gingiva, which can result in necrosis, atrophy, or unusual gingival enlargement.^5^ Atrophy of the gingiva leads to recession and painful sensitivity in the exposed tooth root, whereas gingival enlargement causes food impaction and periodontitis. Based on the fact that ionizing radiation causes physical and chemical tissue damages, some authors suggested that applied RT causes dose-dependent alterations of the vascular flow in dental pulps, with possible but not necessarily permanent consequences: fibrosis and atrophy.^6-8^ Shenoy, et al.^8^ (2007) have reported that the resulting changes in pulp tissue can give a false negative response to standard pulp sensitivity and vitality tests and indicate inadequate treatment: endodontic therapy or even tooth extraction, which can trigger a serious complication: osteoradionecrosis of the jaw.^8,9^

So far, no correlation between the intensity of the acute effects and the severity of late RT sequelae has been proven, which hinders radiation damage protection. The laser Doppler flowmetry (LDF) technique enables non-invasive monitoring of blood flow in different microvascular beds. Analyses of microcirculatory blood flow changes caused by RT at the subclinical level could help in assessing the course and severity of RT side effects. Previous studies have not examined the dependence of microcirculatory parameters on the individual radiation dose received by the corresponding tooth with the adjacent gingiva, which can vary within the total irradiated tissue volume. Therefore, this study aims to determine and compare the dental pulp and gingival blood flow in patients referred for RT of the oral cavity and oropharynx, at three different time points: before the start of RT, immediately after, and six months following the completion of RT, while considering the individual dose received by each tooth and adjacent gingiva. The aim is also to evaluate the dependence of the pulp and gingival blood flow on the received radiation dose.

## Methodology

This study is conducted in accordance with the Declaration of Helsinki. The study protocol was approved by the Ethical Committee of the School of Dental Medicine, University of Belgrade (decision No.36/15, July 8th, 2022). All patients signed an informed consent before enrolling the study.

The study was designed as a prospective study. Data from the literature^10^ were used to estimate the sample size for the difference between the means of the blood flow in gingiva and dental pulp before and after radiation therapy. The sample size consisted of 10 subjects to test the difference between the means for a significance level of 0.05 and a statistical strength of 80% in the program G*Power 3.1.9.4 (Germany).

### Inclusion criteria

1) Patients with a diagnosis of oral/oropharyngeal cancer referred to RT who gave written “informed consent”” to participate in the study
2) Presence of an intact tooth with a healthy adjacent gingiva (without signs of inflammation) and with no history of previous painful sensations

General exclusion criteria:

Presence of any systemic disease that could potentially alter blood flow, such as: arterial hypertension, arryhythmia, peripheral vascular disease, diabetes mellitus, renal, hepatic or respiratory insufficiency, or neurological diseases.

Local exclusion criteria:

1) Discolored tooth
2) Tooth with a history of previous painful sensations
3) Tooth damaged by trauma or caries
4) Tooth with damaged supporting tissues, including gingiva (gingival sulcus depth greater than 2 mm)
5) Tooth reacting painfully to apical palpation and/or percussion

The intact tooth closest to the center of the radiation field was selected for pulp blood flow measurements and the base of interdental papilla of the adjacent gingiva for the gingival blood flow measurements.

All patients were prescribed and received a total radiation dose of 60-66 Gy. The applied modality of therapy in each patient included IMRT. To define the geometry of the photon beams and estimate the volumetric dose distribution in the patient, aiming to precisely irradiate the tumor while sparing the surrounding organs at risk (OAR), Treatment Planning System (TPS) was used. During the planning, the teeth with the adjacent gingiva included in the study were marked. The estimation of the doses for the marked teeth and gingiva was carried out in the TPS, based on the set of data, which allows insight into the geometric-dosimetric parameters of RT at any point in the irradiated tissue volume with defined zones: Gross Tumor Volume (GTV, within the boundaries of gross tumor, i.e. what can be seen, palpated, or imaged), Clinical Target Volume (CTV, shows tumor and surrounding tissues with presumed tumor), Planning Target Volume (PTV, includes CTV and setup margin for patient movements and setup uncertainties), Planning Organ at Risk Volume (PRV, outlines the organs that need to be protected), Treated Volume (TV, provides additional margins around the target volume to allow for limitations of the treatment technique), and Irradiated Volume (IV, the volume of tissue receiving a significant dose).^11^ According to the estimated doses, all the teeth (each of them belonging to one respondent), were divided into two groups: 1) up to 50 Gy, 2) over 50 Gy.

### Laser Doppler flowmetry (LDF)

Blood flow measurements were performed by the laser Doppler flowmeter (PeriFlux PF 5001; Perimed, Jarfalla, Sweden) with red laser light (623.8 nm) using an accompanying software (Perisoft Version 2.50; Perimed, Stockholm, Sweden). Before each blood flow measurement session, the flowmeter was calibrated using a latex particle colloidal suspension (PerimedMotility Standard, Jarfalla, Sweden).

Prior to pulpal blood flow recordings, the probe (407-2, Perimed) was supported on the vestibular tooth surface by a custom-made clear plastic splint (Bioplast; Schen-Dental, Iserlohn, Germany) covering the buccal and lingual surface of the examined tooth and adjacent gingiva. The probe holder (Ph 07-6 Perimed, Jarfalla, Sweden) was incorporated in a plastic splint. The probe was positioned perpendicular to the enamel surface, on the cervical third of the tooth crown, over its central long axis. A rubber dam was applied to isolate the pulp flow from the contribution of the blood flow by the tissues surrounding the tooth.

For gingival blood flow measurements, the probe holder was incorporated in the same plastic splint at the base of the interdental papilla to stabilize the probe perpendicular to the tissue surface and always in the same position.

The blood flow recordings were obtained for at least three minutes and expressed in perfusion units (PU). Measurements were recorded in a quiet room with a constant ambient temperature and by the same dentist after the patient had spent 10 minutes resting in a supine position and after the blood pressure and heart rate had been obtained. The vascular flow was measured on each tooth and adjacent gingiva at three different time points: before the start of RT (FT0), immediately after RT (FT1), and six months following the completion of RT (FT2).

### Statistical analysis

Statistical analyses were conducted in SPSS 22.0 (SPSS Statistics for Windows, SPSS, Inc., Chicago, IL, USA). The values of pulp and gingival flow were expressed as the median. To compare the median flow values measured at the level of the dental pulp and gingiva at three different times, the Friedman test was used. The Wilcoxon test was applied to compare the values obtained within individual-paired time points (FT0 and FT1, FT1 and FT2, FT0 and FT2). Changes in pulpal and gingival flow after the completion of RT (FT1 and FT2) relative to the starting point (FT0) were compared between the groups that received up to 50 Gy and those that received more than 50 Gy, using the Mann-Whitney test. Spearman’s coefficient was used to show the correlation of the radiation dose do the dental pulp and gingival flow. P values < 0.05 were set as significant.

## Results

Due to the two dropouts during the study, eight respondents were included in the analysis (three men and five women), aged 32-68 years, with the average age of 50.75 years (post-hoc achieved power analyzed in G*power program version 3.1.9.2. was 96.29%). All respondents had tumors of the oral cavity, including several different but relatively close localizations: mucosa of the alveolar part of the mandible-1, mucosa of the alveolar part of the maxilla-1, buccal mucosa-1, base of the oral cavity-1, tongue-3, and tongue with the base of the oral cavity-1.

Of a total of eight tested teeth, three were central maxillary incisors, four were lower canines, and one was a lower premolar. Although the total radiation doses in all patients were 60-66 Gy, the individual doses received by the teeth and adjacent gingiva included in the study differed significantly and varied from 20 to 60 Gy. The dose for the tooth and associated gingiva corresponded to the total received dose (60Gy) in only two of eight patients. Four of a total of eight teeth received a dose greater than 50 Gy, whereas the remaining four received a dose of less than 50 Gy.

**Figure 1:**
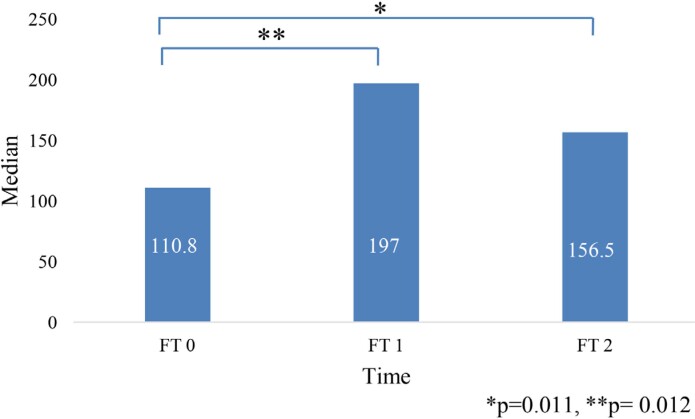
Median value of gingival blood flow measured at three different time points FT0- before the start of RT; FT1- immediately after the completion of RT; FT2- 6 months after the completion of RT.

A descriptive analysis of gingival blood flows showed a significant increase in the median value at FT1 compared to FT0, followed by a decrease at the FT2, although no complete return to the starting point was found (Figure 1). Comparing the gingival flow values obtained at different measurement points, a significant difference (p=0.011) was observed. Additionally, the Wilcoxon test was applied, which showed that the obtained significance comes from the difference in flow values FT0 and FT1 (p=0.012).

**Figure 2:**
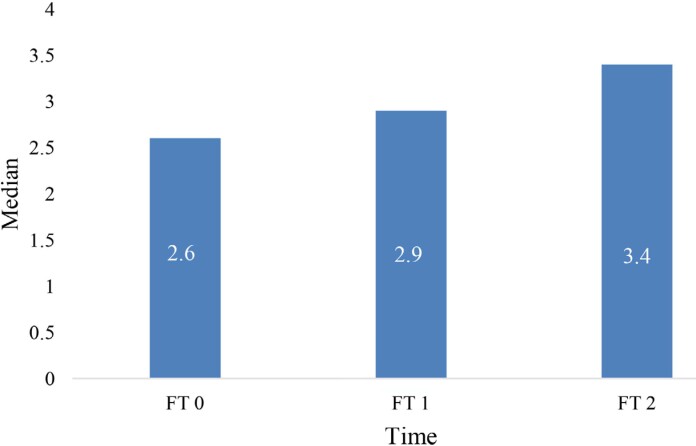
Median value of dental pulp blood flow measured at three different time points FT0- before the start of RT; FT1- immediately after the completion of RT; FT2- 6 months after the completion of RT.

The dental pulp blood flow showed a slight increase of the median value starting from the initial FT0, over the FT1 to FT2 recordings (Figure 2). The median pulp flow values, obtained at experimental times FT1 and FT2, showed no significant difference relative to the median obtained at the initial time FT0 (p=0.325).

**Figure 3:**
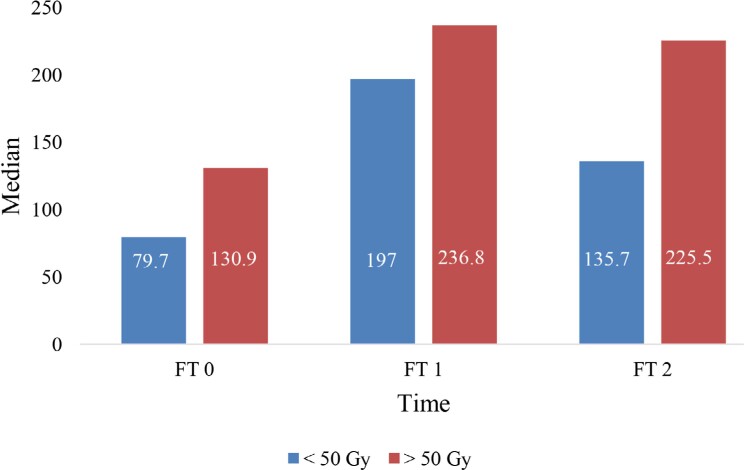
Median value of gingival blood flow at three different time points in relation to the radiation dose FT0- before the start of RT; FT1- immediately after the completion of RT; FT2- 6 months after the completion of RT.

**Figure 4:**
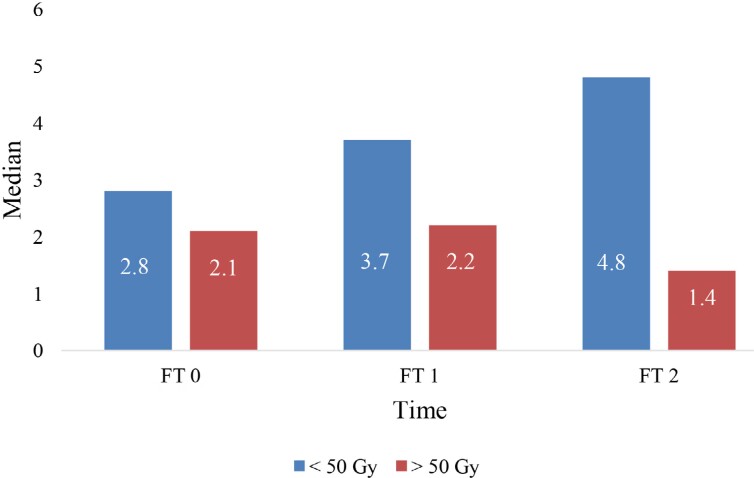
Median value of dental pulp blood flow at three different time points in relation to the radiation dose FT0- before the start of RT; FT1- immediately after the completion of RT; FT2- 6 months after the completion of RT.

The gingival flow values showed an increase after the completion of RT (FT1, FT2) relative to the start point (FT0), regardless of the dose. Still, the increase was higher with the doses >50 Gy, compared with the doses of up to 50 Gy, although without reaching the level of significance (Figure 3). When it comes to the doses > 50 Gy, a decrease in the dental pulp flow rates at FT2 compared to the initial values was observed (FT0) (Figure 4). As opposed to this, regarding the doses of up to 50 Gy, a slight increase in these values prevailed, although without a statistical significance.

The radiation dose and gingival flow showed no statistically significant associations or a strong correlation. In contrast, a negative correlation between the pulp flow and the radiation dose at times FT1 and FT2 was observed, indicating that when the dose is increased, the dental pulp flow decreases (Table 1). Although no statistically significant association was found, a strong correlation was observed six months after the completion of RT (FT2).

**Table 1:** Correlation of the radiation dose to the dental pulp and gingival flow

Radiation dose		
Parameters	Rs	p value
Pulp flow FT1	-0.287	0.490
Pulp flow FT2	-0.575	0.137
Gingival flow FT1	-0.228	0.588
Gingival flow FT2	0.156	0.713

Rs – Spearman correlation coefficient

## Discussion

The result of applied radiotherapy is a dose-dependent obliterant endarteritis that leads to ischemia of soft tissues and fibrosis, whereas the irradiated bone becomes hypovascular and hypoxic.^9,12,13^ The most frequent early side effect of RT applied in the oropharyngeal region is mucositis, which develops in about 80% of patients, and manifests by the atrophy of epithelial cells combined with an inflammatory infiltrate. Its pathogenesis is complex and based on the interactions of various cellular and tissue factors, including the effects of the oral cavity microflora and the development of vascular reactions.^13^ With doses of 50-65 Gy, several months after the end of RT, late effects in the form of soft tissue atrophy and fibrosis can be expected^12^. Based on this, the patients included in our study were divided into two groups according to the received dose at the level of the selected tooth with adjacent gingiva (up to 50 Gy and >50 Gy). These late effects are mainly caused by microvascular damage and manifested as changes in the blood flow or blood vessel diameter and architecture.^12^ Microvascular parameters in the irradiated oral mucosa were analyzed *in vivo* for the first time by Davoudi, et al.^14^ (2013), using the Doppler Optical Coherence Tomography (DOCT) method, which showed that the average speed of the blood flow in all examined regions of the oral mucosa was 1.8 times higher and the pulsation index was 2 times higher in patients after completed RT than in healthy volunteers. In addition, higher values of both flow rate and pulsatility index were recorded in the regions that received a higher RT dose on the side of the tumor (56-66 Gy) compared to the contralateral side (47-53 Gy), although how much time elapsed from the completion of RT to the measurement is not mentioned. A similar trend of a dose-dependent increase in the blood flow after completed RT was recorded for the gingivae in our study, which can be attributed to more pronounced inflammatory changes. Using the same method, Maslennikova, et al.^13^ (2017) detected a significant increase in the total length of blood vessels with a diameter of <15 μm, with a dose of approximately 30 Gy (in the middle of the treatment), before the appearance of any clinical signs of mucositis. This finding indicated the usefulness of DOCT in terms of prediction and early detection of mucositis.^13^ Helmers, et al.^10^ (2017) analyzed microvascular changes of the buccal mucosa and gingiva in patients undergoing RT using the video microscope “Cyto Cam.” The differences observed in the irradiated mucosa referred to telangiectasias, although neither buccal nor gingival mucosa showed significant differences in the microvascular flow index determined before and six months after the completion of RT. The results of our study showed a significant increase of the gingival blood flow at the end of RT compared with the initial values (FT1:FT0), which can be attributed to the inflammatory response. Six months following the RT completion (FT2), the median flow showed a tendency to decrease, but without reaching the initial values (FT0). The accumulation of dental plaque and the burden of microorganisms can affect inflammatory changes and thus the density of gingival capillaries.^10^ A comparison of periodontal indices before starting and six months after the end of RT showed a significant increase in Siller’s oral hygiene index (OHIS), epithelial attachment level (CAL), and gingival recession (GR).^15^ The increase in the oral hygiene index could be explained by the associated hyposalivation, pain, and trismus, but also by the lack of psychological and emotional motivation of oncology patients.^15^ Gingival recession was more pronounced in the mandible than in the maxilla, which can be related to the lesser degree of vascularization. In addition, an unusual enlargement of the gingiva several months after the end of RT was mentioned in the literature, whereas the multiplication of gram-negative bacilli is cited as a possible mechanism for its occurrence.^5^ A change in the oral microflora and an increase in the level of endotoxin released by gram-negative bacilli could play an important role in the development of inflammatory changes in the gingiva and lead to an altered tissue response and consequent gingival enlargement. Poor oral hygiene undoubtedly supports and accelerates the process of periodontium alteration; therefore, dentists must educate their patients undergoing RT.

Although a descriptive analysis of pulp flows in our study showed a slight increase in the median value after completed RT compared to the initial value, no statistically significant difference was observed. Kataoka, et al.^16^ (2011) showed different responses of dental pulp circulation to RT using the pulse oximetry method. The authors observed a significant reduction in the pulp oxygenation level from the initial 93% before treatment to 77% at the end of the RT treatment (60-70Gy), including a repeated increase in %SpO2 values with a tendency to approach initial values (85%) 4-5 months after treatment. These differences could be explained by the fact that the study by Kataoka, et al.^16^ (2011) was based on a different methodology (pulse oximetry against blood flow in our study), differences in the age structure of the examined patients, as well as probable differences in the received dose at the level of the examined teeth. The authors did not evaluate the doses of radiation for the tooth per se. Furthermore, the average age of the patients was 47.2 years (the oldest patient was 55 years old), whereas in our study the average age was 50.75 years (the oldest patient was 68 years old). Our results showed a decrease in the median pulp flow FT2 compared to the initial value of FT0 with doses > 50 Gy; however, an increase in these values prevailed with lower doses, although no statistical significance was found. Correlation results confirmed a negative association between the pulp flow and radiation dose, indicating that increasing the dose decreases the pulp flow, as a strong correlation noted six months after the completion of RT was found. The fine vascular network of the dental pulp, enclosed within the surrounding dentin and without collateral circulation, creates a low compliance circulatory system that is probably more sensitive to high doses of radiation compared to that in the gingiva. Although the studies of Kataoka, et al.^7, 16^ (2016, 2011) showed the recovery of pulp oxygenation few months or years after the completion of RT. The authors did not evaluate the doses of radiation for the tooth per se. Still, in current RT modalities, these doses deviate to a greater or lesser extent from the doses within the Gross Tumor Volume (GTV). Although only in a limited number of cases the dose received by the tooth really corresponds to the general dose, permanent consequences can still be expected. Therefore, regular clinical examinations and tooth vitality testing are certainly recommended during and after RT treatment. Any necrosis of the pulp not treated in time with an endodontic approach may lead to tooth extraction and be complicated by osteoradionecrosis.

As limitation of our study we cite the sample size. Although a difference in the flow changes was observed after the end of therapy between the teeth that received <50 Gy and those that received >50 Gy, statistical significance was not reached. Knowing the fact that the pulp flow showed a strong negative correlation with the radiation dose six months after RT completion, it would be justified to conduct a comparative study of the pulp flow changes after completed RT between the teeth that received <50 Gy and those that received >50 Gy, with a larger sample. The strict exclusion criteria should not be ignored, as well as the fact that this is the first study that considered the variability of radiation doses for the tooth and adjacent gingiva, which is the reality of current RT modalities. The most demanding criterion, which a small number of patients aged 30-70 satisfy, is the presence of intact teeth and healthy gingiva. Consequently, our sample consisted of teeth from different morphotypes (central maxillary incisors, lower canines, and lower premolar) that, according to Norer, et al.^17^ (1999), could potentially affect PBF levels. We believe that this has no significant impact on our results considering the fact that we observed and compared blood flow levels of the same tooth at three-time intervals (before, after, and six months following radiation therapy). The studies by Kataoka, et al.^7,16^ (2016, 2011) also considered teeth with smaller restorations, but we believe that any preparation and production of secondary dentin could affect the vascular flow.

## Conclusion

Currently, highly conformal dose distributions and dose gradients in pathological and normal tissues, delivered by IMRT, have made the appearance, severity, and possible sequelae specific to each patient, requiring a strictly individual approach.

In gingivae, RT causes acute blood flow increase, followed by a long-term (over six months) tendency to return to the starting levels, whereas the dental pulp blood flow is differently affected by higher radiation doses (over 50 Gy) in comparison to lower doses (below 50 Gy).

When planning RT, we recommend considering the possibility of protecting the teeth localized along the GTV as a sensitive organ to avoid permanent consequences, especially if the given teeth are perceived as potential carriers of future prosthetic restorations. Before starting RT, we advise a consultation with a dentist and, in indicated cases, pre-irradiation dental treatment.
